# Mirabegron 50 mg once daily, long-term treatment maximizes benefit in middle-aged and older people with overactive bladder syndrome: a systematic review and meta-analysis of nine phase II/III, randomized, double-blind, parallel-design, placebo-controlled, multicenter, and multinational trials

**DOI:** 10.3389/fsurg.2024.1372175

**Published:** 2024-08-26

**Authors:** Xiangxiang Zhang, Yinhui Mao, Yang Liu, Jilei Sun, Juntao Sun, Chenli Pan, Zhuo Wang, Zhitao Wei, Yong Yang

**Affiliations:** ^1^ Changchun University of Chinese Medicine, Changchun, China; ^2^ Department of Urology, The Affiliated Hospital of Changchun University of Chinese Medicine, Changchun, China

**Keywords:** meta-analysis, mirabegron, placebo, randomized controlled trials, urinary bladder, overactive, middle-aged and older people

## Abstract

The prevalence and severity of overactive bladder increase with age, and mirabegron is an approved treatment for this condition. This meta-analysis systematically evaluated the efficacy and safety of mirabegron compared with placebo for overactive bladder treatment. We searched PubMed and the Cochrane Library (30 October 2023) for relevant articles (source: MEDLINE, EMBASE, ClinicalTrials.gov, ICTRP, CINAHL). We included randomized controlled trials involving adults with overactive bladder syndrome that compared mirabegron with placebo treatment. Data were analyzed according to the Cochrane Handbook for Systematic Reviews of Interventions [Review Manager (computer program) Version 5.4]. Nine parallel-group trials (10 articles) were included. The evaluation included a total of 8,527 adults, including 6,445 women and 2,082 men, of whom 5,726 were White, 2,462 were Asian, and 161 were Black. The mean age of the participants ranged from 53.4 to 60.3 years. This evaluation involved three specifications of mirabegron: 25 mg, 50 mg, and 100 mg. In all trials, patients were enrolled in a 12-week double-blind treatment period, and the dose was once daily. The review of trials found that on average, people taking mirabegron had about 13 ml more volume voided per micturition, five fewer micturitions, and four fewer incontinence episodes every week, with moderate improvements in quality of life. About one in five people taking the drug reported TRAEs. Mirabegron treatment is well tolerated, with the risk of adverse events similar to that of a placebo. For best results, a dose of 50 mg once daily is recommended for long-term use. It is unclear whether any benefits are sustained after treatment discontinuation.

**Systematic Review Registration**: https://www.crd.york.ac.uk/prospero/, PROSPERO (CRD42023430737).

## Background

The International Continence Society (ICS) defines overactive bladder (OAB) as a bladder storage symptom syndrome: “urgency, with or without urgency urinary incontinence, usually with increased daytime frequency and nocturia” (1). Urgency is a sudden and strong urge to urinate that is difficult to postpone, and sometimes there is involuntary urinary leakage, called urgency urinary incontinence. Urinating more than eight times in a 24 h period is recognized as frequent in clinical practice. If a person wakes up over once during the nighttime to urinate from asleep, the condition is known as nocturia (2). In 2008, the prevalence of OAB was approximately 10.7% of the global population of 4.3 billion. It was previously estimated that by 2018, 546 million people would be affected by OAB (20.1%) (3). As a highly prevalent disease, the prevalence and severity of OAB increase with age (4, 5). As the world is expected to enter an aging society, OAB results in adverse effects on patients’ health-related quality of life and a significant financial burden, on the one hand, and may put increasing pressure on healthcare resources, on the other hand (6–9). The myogenic and urothelial-neurogenic hypotheses are the two most frequently recognized explanations for OAB, which is caused by multiple underlying pathophysiologic mechanisms and should be viewed as a complex, multifactorial symptomatic syndrome (10). Current treatment options for OAB include behavioral therapy, pharmacotherapy, minimally invasive surgery, and other surgical options (11). Clinical guidelines identified behavioral therapy with or without pharmacotherapy as the first-line treatment and pharmacotherapy alone as the second-line therapy for OAB (12). This evaluation's focus is solely on pharmaceutical care.

One of the main pharmacologic treatments for OAB is to block the binding of acetylcholine to muscarinic receptors in the bladder wall with anticholinergic drugs; the intestines, salivary glands, eyes, brain, and other areas of the body do, however, have muscarinic receptors. Consequently, this category of medications can have negative effects on several physiological systems, such as constipation, dry mouth, blurred vision, and cognitive dysfunction (13–15). These side effects cause some patients to become intolerant and discontinue treatment, and they particularly hinder the durability of treatment for middle-aged and elderly OAB patients whose base medication is in this class. Mirabegron is a β_3_-adrenergic receptor agonist that selectively stimulates bladder β_3_-adrenergic receptors, mediates relaxation of the detrusor, and modulates sensory pathways, bladder afferent neural activity, and neurotransmitter release, from the urothelium, thereby increasing bladder capacity and decreasing bladder sensitivity to alleviate the storage-phase symptom syndrome—OAB (10, 16, 17). At the same time, it has been shown that mirabegron has a concentration-dependent diastolic effect on the detrusor, which results from a combination of action through agonism of β_3_-adrenergic receptors and antagonism of α_1_-adrenergic receptors (18). It was approved by the US Food and Drug Administration in 2012 for the treatment of OAB symptoms and is an alternative treatment regimen for antimuscarinic treatment of OAB (19). To support and further define the reported efficacy and safety of adult patients receiving mirabegron monotherapy, we included evidence from the most recent extant global clinical trials of 12-week placebo-controlled randomized studies in patients with OAB. We aimed to integrate these existing high-level studies and conduct a meta-analysis of these studies to explore mirabegron for OAB efficacy and safety.

## Objectives

To evaluate the efficacy of mirabegron in the treatment of overactive bladder syndrome in comparison to a placebo. We will address the following assumption: mirabegron is more effective than a placebo in managing overactive bladder syndrome.

## Methods

### Criteria for considering studies for this review

#### Types of studies

All randomized controlled trials of mirabegron vs. placebo of overactive bladder syndrome.

#### Types of participants

All adult males and females who have been diagnosed with overactive bladder syndrome according to symptoms.

#### Types of interventions

In one study, mirabegron had to be used in at least one research arm, while the other arm was a placebo. The medication has to be administered to lessen the symptoms of an overactive bladder.

#### Types of outcome measures

The indicators of the outcome, objective as well as subjective, were incorporated in this evaluation.

### Primary outcomes

Quantification of symptoms: volume voided per micturition, micturitions in 24 h, and incontinence episodes in 24 h.

### Secondary outcomes

A. Patient's satisfaction scores with treatments: TS-VAS, PPBC, and OAB-q.
B. Adverse events: TRAEs and TEAEs.

### Search methods for identification of studies

We did not impose any language or other restrictions on any of the searches.

### Electronic searches

The latest search for this evaluation was conducted on 30 October 2023. We searched PubMed and the Cochrane Library; the relevant articles were obtained from databases including MEDLINE, EMBASE, ClinicalTrials.gov, ICTRP, and CINAHL. Relevant trials were identified from the Cochrane Central Register of Controlled Trials (CENTRAL), which is regularly updated with the Cochrane Library. The evaluation has drawn on the Cochrane Collaboration's recommendation to use a highly sensitive search strategy specifically for MEDLINE randomized controlled trials using the Pubmed search route.

The search terms and strategies used are presented in Table 1.

**Table 1:** Search terms and strategies used.

#1	“Urinary Bladder, Overactive”[Mesh]
#2	(Overactive Bladder[Title/Abstract) OR (Overactive Urinary Bladder[Title/Abstract) OR (Bladder, Overactive[Title/Abstract) OR (Overactive Detrusor[Title/Abstract) OR (Detrusor, Overactive[Title/Abstract) OR (Overactive Detrusor Function[Title/Abstract) OR (Detrusor Function, Overactive[Title/Abstract)
#3	(((((((“Urinary Bladder, Overactive”[Mesh) OR (Overactive Bladder[Title/Abstract)) OR (Overactive Urinary Bladder[Title/Abstract)) OR (Bladder, Overactive[Title/Abstract)) OR (Overactive Detrusor[Title/Abstract)) OR (Detrusor, Overactive[Title/Abstract)) OR (Overactive Detrusor Function[Title/Abstract)) OR (Detrusor Function, Overactive[Title/Abstract)
#4	“mirabegron” [Supplementary Concept]
#5	(Betmiga[Title/Abstract) OR (2-(2-aminothiazol-4-yl)-4′-(2-((2-hydroxy-2-phenylethyl)amino)ethyl)acetanilide[Title/Abstract) OR (Betanis[Title/Abstract) OR (YM 178[Title/Abstract) OR (YM-178[Title/Abstract)
#6	(((((“mirabegron” [Supplementary Concept) OR (Betmiga[Title/Abstract)) OR (2-(2-aminothiazol-4-yl)-4′-(2-((2-hydroxy-2-phenylethyl)amino)ethyl)acetanilide[Title/Abstract)) OR (Betanis[Title/Abstract)) OR (YM 178[Title/Abstract)) OR (YM-178[Title/Abstract)
#7	(((((((randomized controlled trial[pt) OR (controlled clinical trial[pt)) OR (randomized[tiab)) OR (placebo[tiab)) OR (drug therapy [sh)) OR (randomly[tiab)) OR (trial [tiab)) OR (groups[tiab)
#8	(((((((((“Urinary Bladder, Overactive"[Mesh) OR (Overactive Bladder[Title/Abstract)) OR (Overactive Urinary Bladder[Title/Abstract)) OR (Bladder, Overactive[Title/Abstract)) OR (Overactive Detrusor[Title/Abstract)) OR (Detrusor, Overactive[Title/Abstract)) OR (Overactive Detrusor Function[Title/Abstract)) OR (Detrusor Function, Overactive[Title/Abstract)) AND ((((((“mirabegron” [Supplementary Concept) OR (Betmiga[Title/Abstract)) OR (2-(2-aminothiazol-4-yl)-4′-(2-((2-hydroxy-2-phenylethyl)amino)ethyl)acetanilide[Title/Abstract)) OR (Betanis[Title/Abstract)) OR (YM 178[Title/Abstract)) OR (YM-178[Title/Abstract))) AND ((((((((randomized controlled trial[pt) OR (controlled clinical trial[pt)) OR (randomized[tiab)) OR (placebo[tiab)) OR (drug therapy [sh)) OR (randomly[tiab)) OR (trial [tiab)) OR (groups[tiab))

### Searching other resources

The reference list of relevant articles was searched for other potentially relevant trials.

### Data collection and analysis

#### Selection of studies

Without first taking into account their outcomes, both researchers separately evaluated the appropriateness of the trials that were under consideration for inclusion in this study. A third party evaluated any disagreements that could not be settled through discussion. The excluded studies and their reasons for exclusion are listed.

### Data extraction and management

The data were extracted and cross-checked independently by at least two researchers. Further explanation was requested from the researchers in cases where data were gathered but not reported or presented in a way that was suitable for incorporation in the formal evaluation.

### Assessment of risk of bias in included studies

The researchers independently assessed the risk of bias using the Cochrane Collaboration Network's risk of bias assessment tool, which includes random sequence generation, allocation concealment, blinding of participants and personnel, blinding of outcome assessment, incomplete outcome data, selective reporting, and other biases. Disagreements were resolved by discussion with a third party.

### Measures of treatment effect

In accordance with the Cochrane Handbook for Systematic Reviews of Interventions, data from included trials were handled. For dichotomous data, the Mantel–Haenszel fixed-effect approach was used to calculate the risk ratio as the effect measure; for continuous data, the inverse variance fixed-effect method was used to calculate the mean difference. In the meta-analysis, data from trials reporting changes in end-of-treatment scores compared to baseline scores were merged.

### Unit of analysis issues

Data from all trials must be given as the mean and standard deviation of the difference from the baseline of two treatments for continuous data to be used in this evaluation, as the correlation between measurements on the same individual may be important.

### Data synthesis

The indicators of targeted results from the included studies were combined in this formal evaluation, if appropriate, to produce an overall estimate of the treatment effect using a fixed-effect model.

### Subgroup analysis and investigation of heterogeneity

The subgroup analyses were planned to investigate the effects of the dose. The clinical and methodological heterogeneity of the studies was assessed. To check for signs of statistical dissimilarity in the data plots, a statistical test for heterogeneity was applied. If heterogeneity was noticed, an explanation was looked for and described in the article (based on the *I*^2^ statistic and the test for heterogeneity). The data were analyzed after the trials that were the source of the discrepancy were removed from all data plots where three or more trials were involved.

### Sensitivity analysis

By removing trials that resulted in considerable heterogeneity at a certain dose, the analysis of sensitivity was carried out. The article just reported the findings after the analysis of sensitivity.

## Results

### Description of studies

See “Characteristics of included studies” and “Characteristics of excluded studies” in the Appendix.

### Results of the search

The search yielded 525 records, which were then vetted for eligibility; 55 full-text articles were acquired.

### Included studies

**Figure 1:**
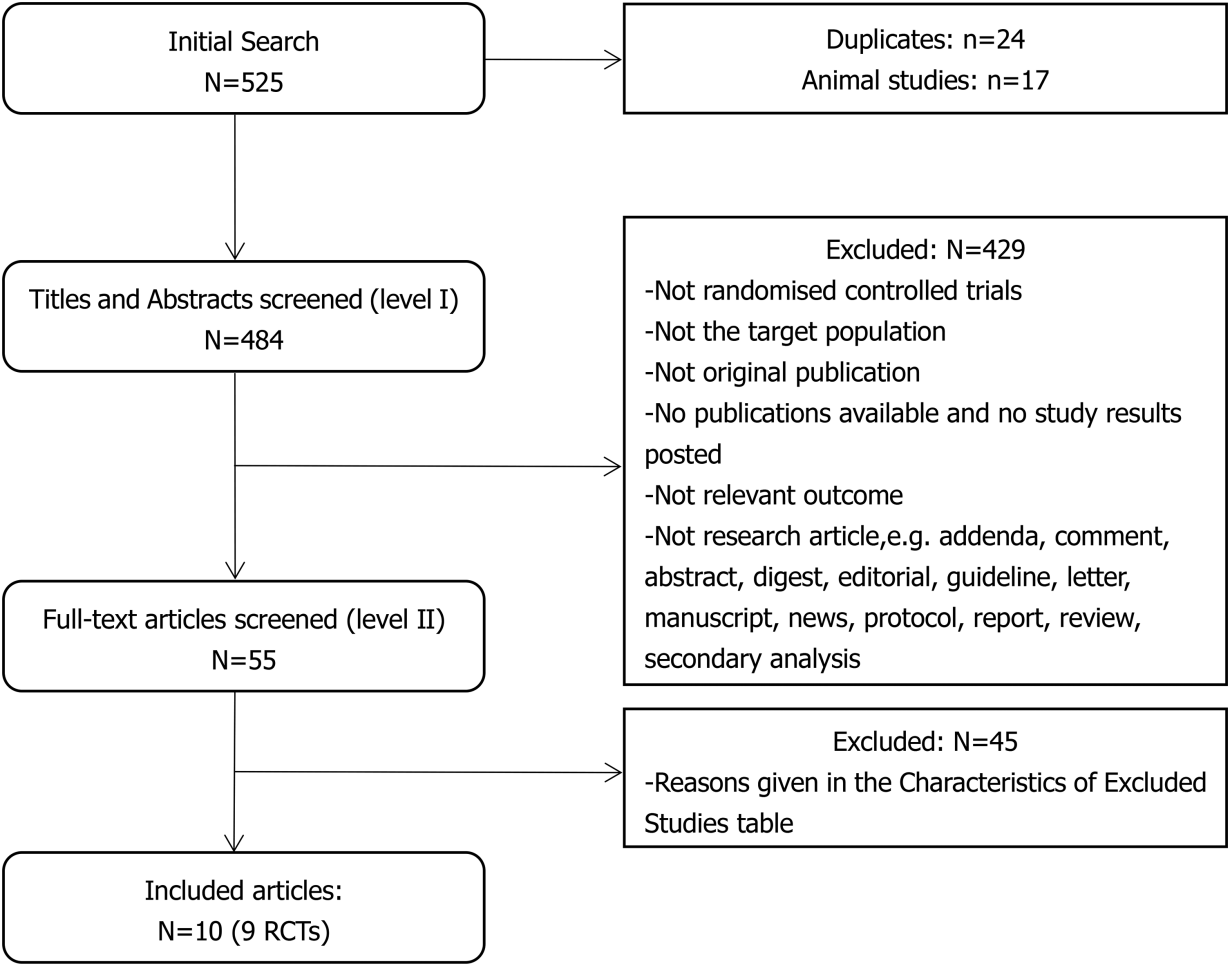
Study flow diagram.

Ten independent reports (20–29) of nine randomized controlled trials were included in the evaluation, all with a parallel design. Figure 1 shows the flow of literature through the assessment process. The evaluation examined only that part of all reports in which mirabegron was compared with placebo and made one type of comparison: comparisons of different doses (25 mg, 50 mg, and 100 mg) of mirabegron vs. placebo. All trials were given at a once-daily dose. Sample sizes ranged from 236 (20) to 1,483 (25).

The trials included people ≥18 years old with symptoms of overactive bladder (OAB) for ≥3 months and a diagnosis of OAB met after assessment of a 3-day urinary diary. Exclusion criteria were clearly defined for all but one report (20), where the exclusion criteria were unclear. The evaluation included a total of 8,527 adults, including 6,445 women (∼76%) and 2,082 men (∼24%), of whom 5,726 were White (67%), 2,462 were Asian (29%), and 161 were Black (about 2%). The mean age of the participants ranged from 53.4 to 60.3 years, and the standard deviation ranged from 11.84 to 14.5. In many trials, patients were enrolled in a single-blind, 2-week placebo run-in period, followed by a 12-week double-blind treatment period. In one trial, treatment was preceded by a 4-week placebo run-in period (23).

Overall, there was inconsistency in the sorts of outcome measures provided by trialists as well as in the way data were recorded. The primary outcomes of the target in the evaluation were the quantification of symptoms, including volume voided per micturition, micturitions in 24 h, and incontinence episodes in 24 h. Another quantitative measure that was one of the most usually reported secondary outcomes of the target was patient observations (e.g., perception of cure or improvement), which included the TS-VAS, PPBC, and OAB-q. For continuous data, the mean and standard deviation of the difference from baseline between two treatments were statistical and calculated to incorporate these data into the evaluation. In this manner, 10 independent reports of nine parallel trials supplied data (20–29). The other most usually reported secondary outcome of the target was adverse events, such as TRAEs and TEAEs. The data must be presented in the evaluation as a two-by-two table for binary data (20, 21, 23, 26, 28, 29).

### Risk of bias in included studies

The generation of random allocation, concealment of allocation, blinding of trial participants and investigators, completeness of treatment, withdrawals and dropouts, and loss to follow-up were examined to evaluate the methodological quality of the published studies.

### Randomization, allocation concealment, and blinding

**Figure 2:**
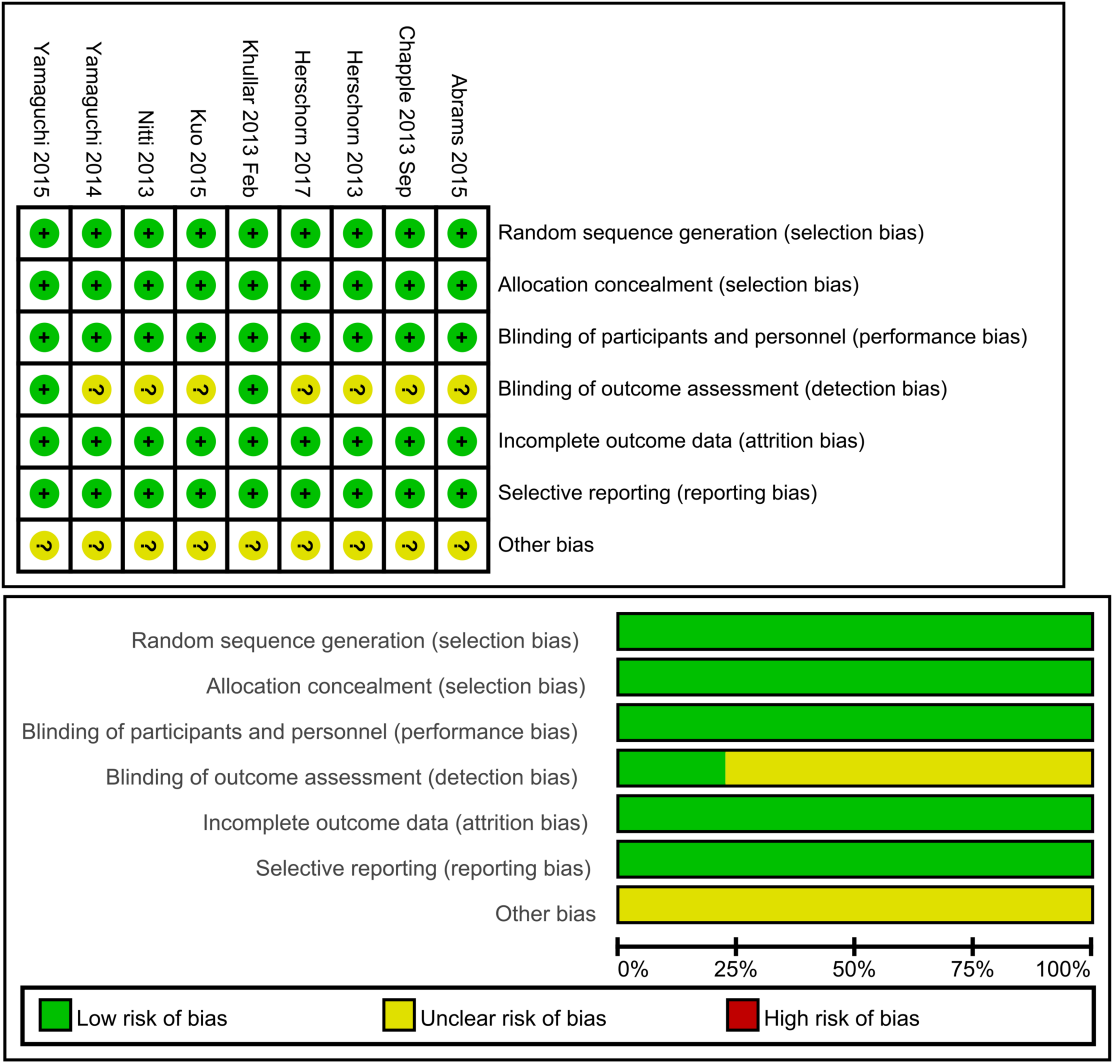
Risk of bias summary and graph: review authors’ judgments about each risk of bias item for each included study.

Rarely was the grouping procedure described. Although group allocation should be sufficiently concealed by double blinding, this is not a given. Trials that declared group allocation was “double-blind” were categorized as having adequate concealment for the evaluation. In the nine trials (20–23, 25–29), it was known that allocation was sufficiently concealed. Although the nine trials were double-blinded, only two trials specifically stated that outcome assessors were blind to group allocation (25, 29). Some studies stated that the code was broken at the completion of the study, and in some, it was specified that this was after the analysis. This would imply that the final measurement was done blind. Consequently, the evaluation has been considered to have sufficient allocation concealment. All nine parallel-group trials claimed that the groups were comparable at baseline. The risk of bias summary and graph are shown in Figure 2.

### Withdrawals and dropouts

The reasons for discontinuation were mentioned in all trials. The dropout rate in four trials was 10% or less (21, 25, 28, 29). One trial did not state the number of dropouts in each group, so the dropout rate was not sure (20). The dropout rates in the remaining trials varied in parallel designs from 11% (23) to 21% (26). More than half of the parallel-design trials included any follow-up. Spans of time, such as 2 weeks (20, 26, 28) or 4 weeks (25, 27), were used in the trials that did follow-up individuals.

### Effects of interventions

Comparison 1. Mirabegron versus placebo. The data is presented in Table 2.

**Table 2:** Mirabegron versus placebo.

Outcome or subgroup	Studies	Participants	Statistical method	Effect estimate
1.1 Mean volume voided per micturition	9	10,882	Mean difference (IV, fixed, 95% CI)	12.50 (10.72, 14.28)
1.1.1 Mirabegron 25 mg	5	2,548	Mean difference (IV, fixed, 95% CI)	7.51 (3.58, 11.44)
1.1.2 Mirabegron 50 mg	9	5,780	Mean difference (IV, fixed, 95% CI)	13.41 (11.08, 15.75)
1.1.3 Mirabegron 100 mg	4	2,554	Mean difference (IV, fixed, 95% CI)	14.78 (10.94, 18.62)
1.2 Micturitions in 24 h	8	10,580	Mean difference (IV, fixed, 95% CI)	−0.60 (−0.70, −0.50)
1.2.1 Mirabegron 25 mg	4	2,394	Mean difference (IV, fixed, 95% CI)	−0.51 (−0.72, −0.29)
1.2.2 Mirabegron 50 mg	8	5,631	Mean difference (IV, fixed, 95% CI)	−0.61 (−0.75, −0.48)
1.2.3 Mirabegron 100 mg	4	2,555	Mean difference (IV, fixed, 95% CI)	−0.67 (−0.88, −0.45)
1.3 Incontinence episodes in 24 h	8	8,287	Mean difference (IV, fixed, 95% CI)	−0.47 (−0.56, −0.38)
1.3.1 Mirabegron 25 mg	4	1,954	Mean difference (IV, fixed, 95% CI)	−0.48 (−0.67, −0.30)
1.3.2 Mirabegron 50 mg	8	4,240	Mean difference (IV, fixed, 95% CI)	−0.45 (−0.57, −0.33)
1.3.3 Mirabegron 100 mg	4	2,093	Mean difference (IV, fixed, 95% CI)	−0.50 (−0.69, −0.31)
1.4 TS-VAS	4	3,350	Mean difference (IV, fixed, 95% CI)	0.78 (0.59, 0.97)
1.5 PPBC	3	2,559	Mean difference (IV, fixed, 95% CI)	−0.14 (−0.25, −0.03)
1.6 OAB-q	5	5,729	Mean difference (IV, fixed, 95% CI)	−4.31 (−5.49, −3.13)
1.6.1 Mirabegron 25 mg	3	1,937	Mean difference (IV, fixed, 95% CI)	−2.64 (−4.88, −0.40)
1.6.2 Mirabegron 50 mg	5	3,792	Mean difference (IV, fixed, 95% CI)	−4.95 (−6.33, −3.56)
1.7 TRAEs	6	5,028	Risk ratio (M-H, fixed, 95% CI)	1.12 (0.99, 1.26)
1.7.1 Mirabegron 25 mg	4	1,770	Risk ratio (M-H, fixed, 95% CI)	1.14 (0.91, 1.42)
1.7.2 Mirabegron 50 mg	6	3,258	Risk ratio (M-H, fixed, 95% CI)	1.11 (0.96, 1.28)
1.8 TEAEs	5	4,338	Risk ratio (M-H, fixed, 95% CI)	0.98 (0.91, 1.05)

#### Primary outcome measures: quantification of symptoms, for example, volume voided per micturition, micturitions in 24 h, and incontinence episodes in 24 h (Outcomes 1.1–1.3)

**Figure 3:**
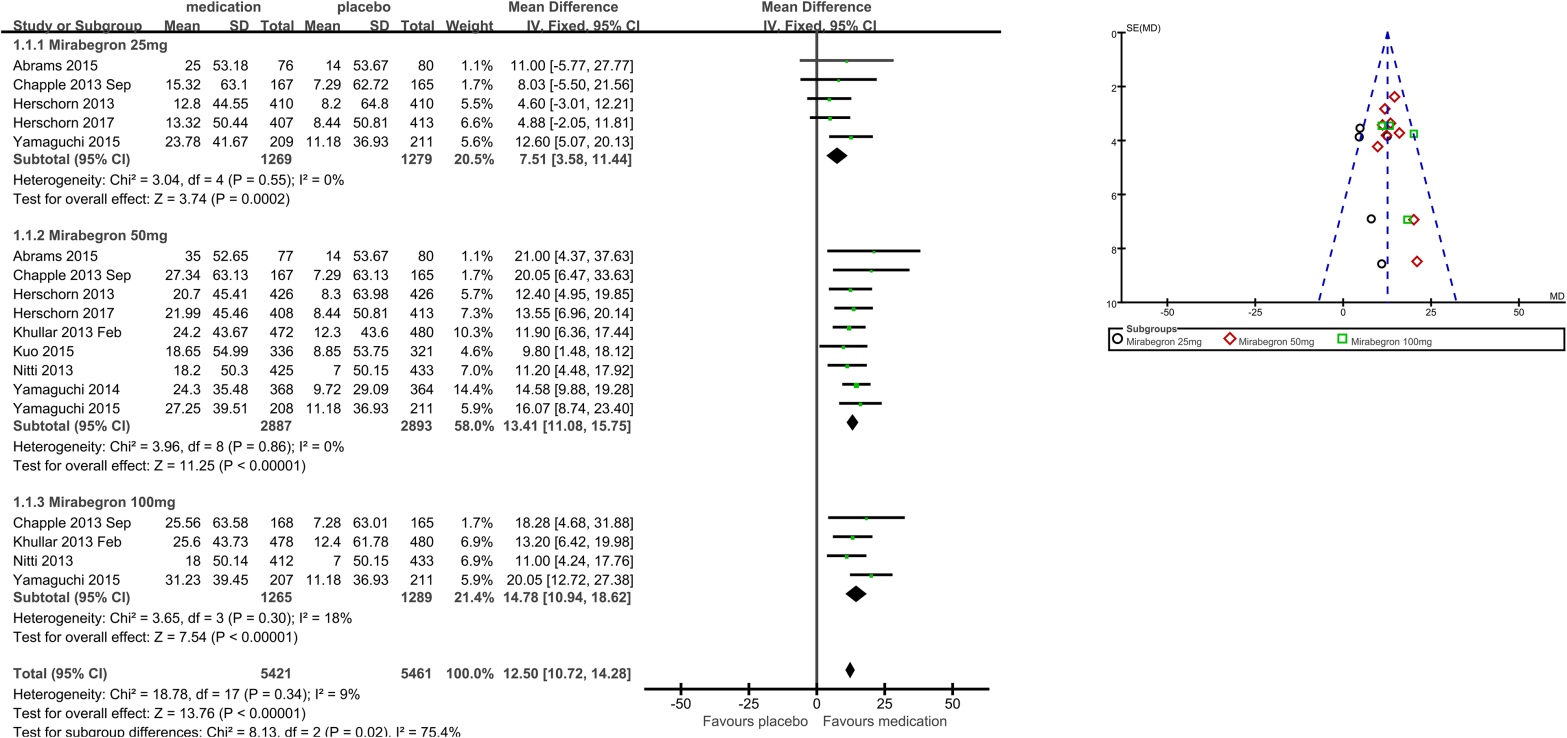
Comparison 1. Mirabegron versus placebo, Outcome 1.1, volume voided per micturition.

Nine trials (20–23, 25–29) reported available data on volume voided per micturition after treatment (Figure 3). Those in the mirabegron groups had approximately 12.50 volume voided more per micturition than those taking placebo (MD for volume voided per micturition 12.50, 95% CI 10.72–14.28, *P* < 0.00001, Outcome 1.1).

#### Mirabegron 25 mg vs. placebo

Five trials (20–23, 29) reported available data on volume voided per micturition after treatment. Those in the mirabegron 25 mg groups had approximately 7.51 volume voided more per micturition than those taking placebo (MD for volume voided per micturition 7.51, 95% CI 3.58–11.44, *P* = 0.0002, Outcome 1.1.1).

#### Mirabegron 50 mg vs. placebo

Nine trials (20–23, 25–29) reported available data on volume voided per micturition after treatment. Those in the mirabegron 50 mg groups had approximately 13.41 volume voided more per micturition than those taking placebo (MD for volume voided per micturition 13.41, 95% CI 11.08–15.75, *P* < 0.00001, Outcome 1.1.2).

#### Mirabegron 100 mg vs. placebo

Four trials (21, 25, 27, 29) reported available data on volume voided per micturition after treatment. Those in the mirabegron 100 mg groups had approximately 14.78 volume voided more per micturition than those taking placebo (MD for volume voided per micturition 14.78, 95% CI 10.94–18.62, *P* < 0.00001, outcome 1.1.3).

**Figure 4:**
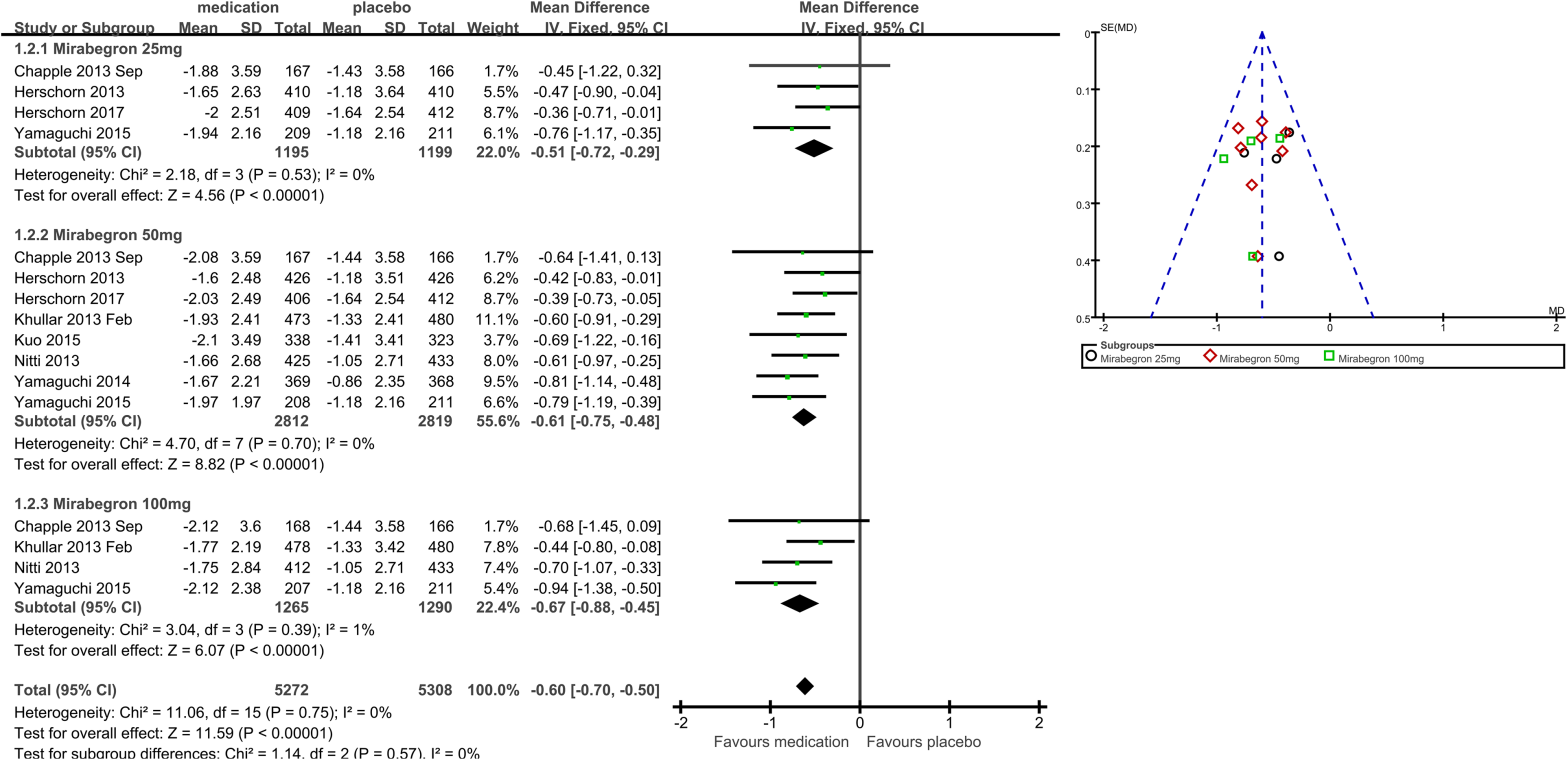
Comparison 1. Mirabegron versus placebo, Outcome 1.2, micturitions in 24 h.

Eight trials (21–23, 25–29) reported available data on micturitions in 24 h after treatment (Figure 4). The number of micturitions per 24 h was roughly 0.60 less in the mirabegron groups than that in the placebo groups (MD for micturitions within a day −0.60, 95% CI −0.70 to −0.50, *P* < 0.00001, Outcome 1.2). The outcome reveals a weekly reduction in micturitions of about five on average.

#### Mirabegron 25 mg vs. placebo

Four trials (21–23, 29) reported available data on micturitions in 24 h after treatment. Approximately 0.51 fewer micturitions per 24 h were made by those using 25 mg mirabegron compared to those receiving a placebo (MD for micturitions within a day −0.51, 95% CI −0.72 to −0.29, *P* < 0.0001, Outcome 1.2.1).

#### Mirabegron 50 mg vs. placebo

Eight trials (21–23, 25–29) reported available data on micturitions in 24 h after treatment. Approximately 0.61 fewer micturitions per 24 h were made by those using mirabegron 50 mg compared to those receiving a placebo (MD for micturitions within a day −0.61, 95% CI −0.75 to −0.48, *P* < 0.00001, Outcome 1.2.2).

#### Mirabegron 100 mg vs. placebo

Four trials (21, 25, 27, 29) reported available data on micturitions in 24 h after treatment. Approximately 0.67 fewer micturitions per 24 h were made by those using mirabegron 100 mg compared to those receiving a placebo (MD for micturitions within a day −0.67, 95% CI −0.88 to −0.45, *P* < 0.00001, Outcome 1.2.3).

**Figure 5:**
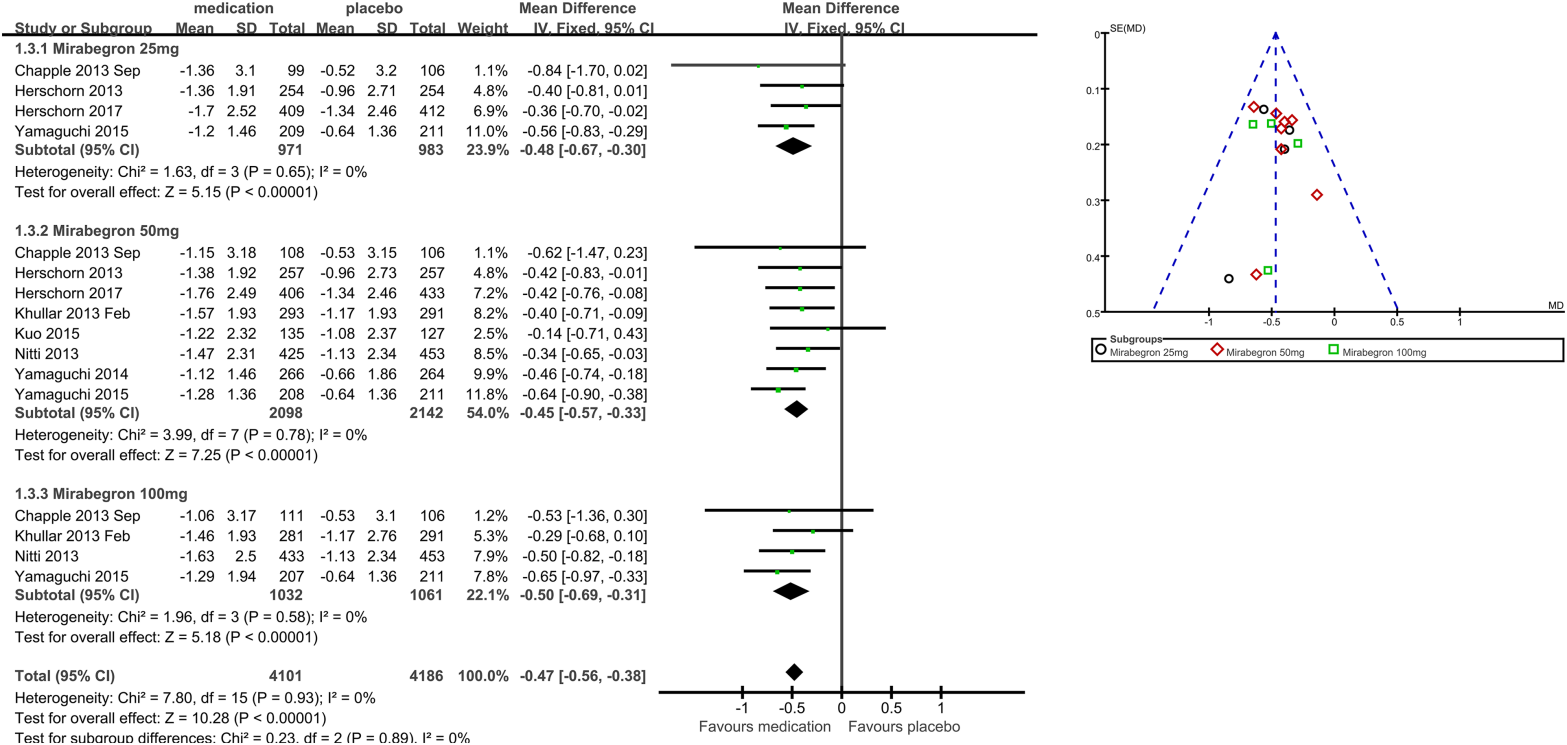
Comparison 1. Mirabegron versus placebo, Outcome 1.3, incontinence episodes in 24 h.

Eight trials (21, 22, 25–29) reported available data on incontinence episodes within 24 h after treatment (Figure 5). The number of incontinence episodes per 24 h was roughly 0.47 less in the mirabegron groups than in the placebo groups (MD for incontinence episodes within a day −0.47, 95% CI −0.56 to −0.38, *P* < 0.00001, Outcome 1.3). The outcome reveals a weekly reduction in incontinence episodes of about four on average.

#### Mirabegron 25 mg vs. placebo

Four trials (21–23, 29) reported available data on incontinence episodes in 24 h after treatment. There were almost 0.48 fewer incontinence events per 24 h in the mirabegron 25 mg groups than in the placebo groups (MD for incontinence episodes within a day −0.48, 95% CI −0.67 to −0.30, *P* < 0.00001, Outcome 1.3.1).

#### Mirabegron 50 mg vs. placebo

Eight trials (21, 22, 25–29) reported available data on incontinence episodes in 24 h after treatment. There were roughly 0.45 fewer incontinence incidents per 24 h in the mirabegron 50 mg groups than in the placebo groups (MD for incontinence episodes within a day −0.45, 95% CI −0.57 to −0.33, *P* < 0.00001, Outcome 1.3.2).

#### Mirabegron 100 mg vs. placebo

Four trials (21, 25, 27, 29) reported available data on incontinence episodes in 24 h after treatment. There were roughly 0.50 fewer incontinence events per 24 h in the mirabegron 100 mg groups than in the placebo groups (MD for incontinence episodes within a day −0.50, 95% CI −0.69 to −0.31, *P* < 0.00001, Outcome 1.3.3).

### Secondary outcome measures: patient observations, for example, TS-VAS, PPBC, and OAB-q (Outcomes 1.4–1.6)

**Figure 6:**
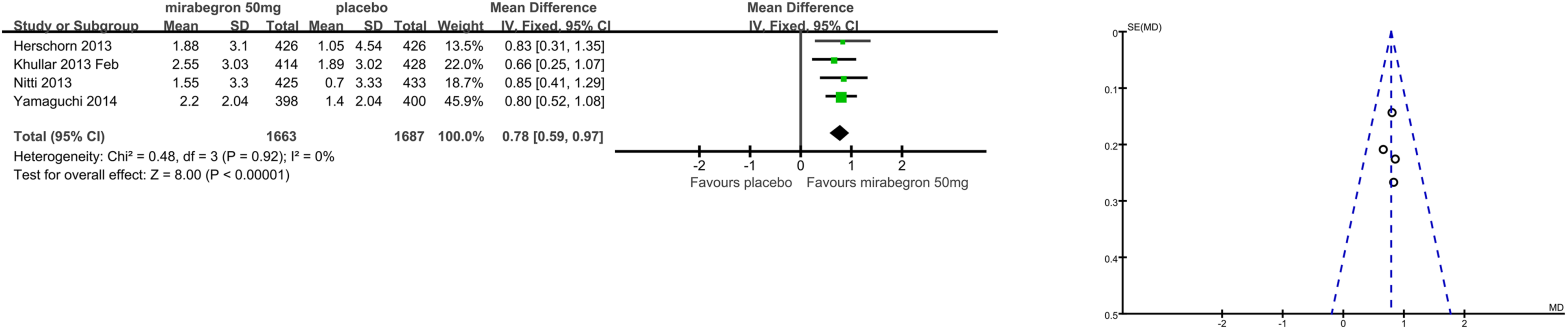
Comparison 1. Mirabegron versus placebo, Outcome 1.4, TS-VAS.

**Figure 7:**
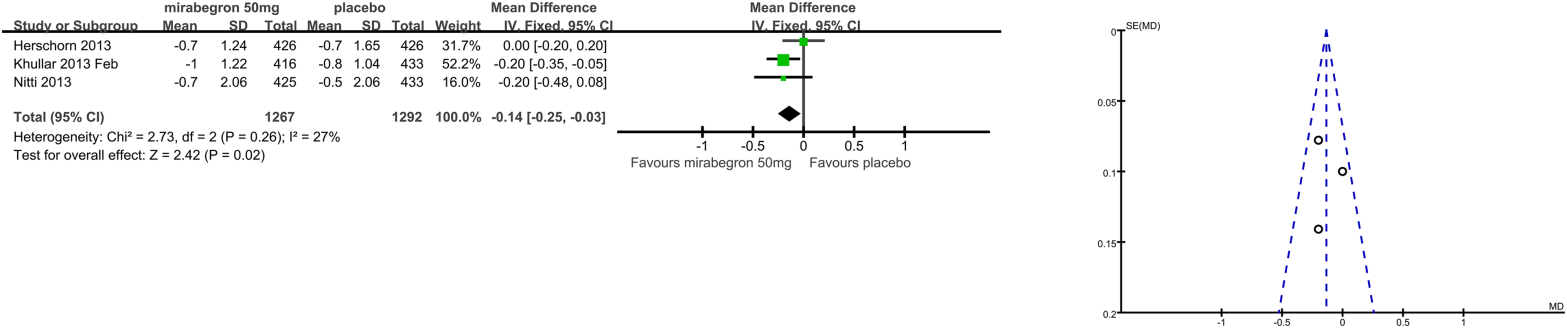
Comparison 1. Mirabegron versus placebo, Outcome 1.5, PPBC.

**Figure 8:**
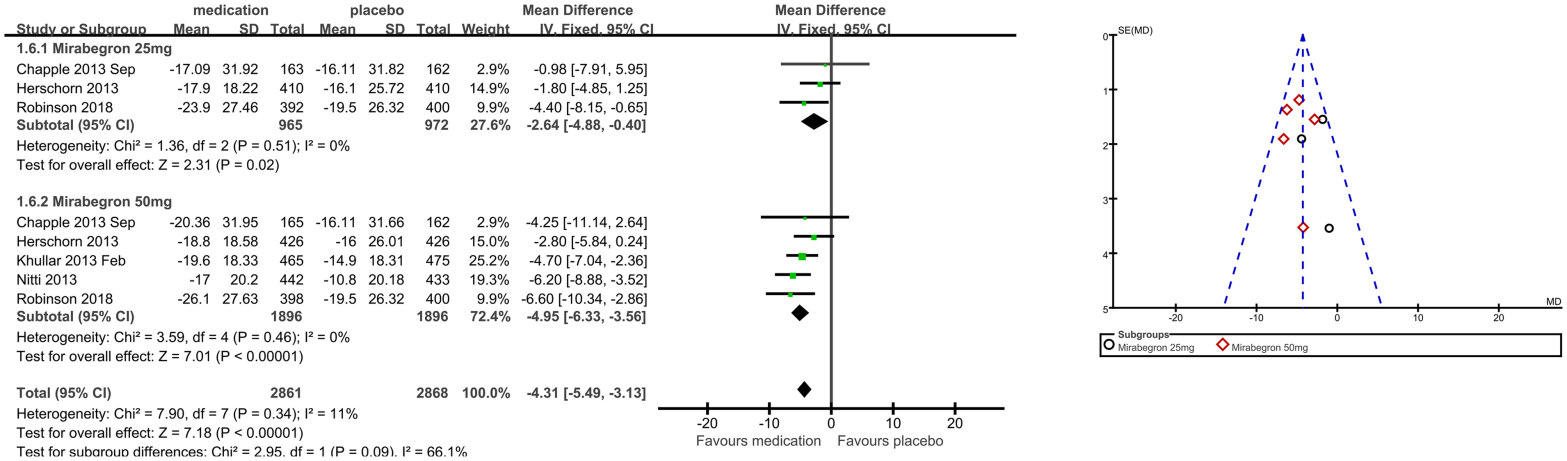
Comparison 1. Mirabegron versus placebo, Outcome 1.6, OAB-q.

Patients’ perceptions of change including TS-VAS, PPBC, and OAB-q were reported in five articles (21, 22, 24, 25, 27). Those taking medication had a higher likelihood of attesting to a cure or an improvement in their symptoms than those receiving a placebo, mean difference (MD) for TS-VAS (Figure 6), 0.78 (95% CI 0.59–0.97, *P* < 0.00001, Outcome 1.4); MD for PPBC (Figure 7), −0.14 (95% CI −0.25 to −0.03, *P* = 0.02, Outcome 1.5); MD for OAB-q (Figure 8), −4.31 (95% CI −5.49 to −3.13, *P* < 0.00001, Outcome 1.6).

Three articles (21, 22, 24) reported available data for mirabegron 25 mg in OAB-q (MD −2.64, 95% CI −4.88 to −0.40, *P* = 0.02, Outcome 1.6.1). Five articles (21, 22, 24, 25, 27) reported available data for mirabegron 50 mg in OAB-q, with a statistically significant difference (MD −4.95, 95% CI −6.33 to −3.56, *P* < 0.00001, Outcome 1.6.2).

### Adverse events (Outcomes 1.7–1.8)

**Figure 9:**
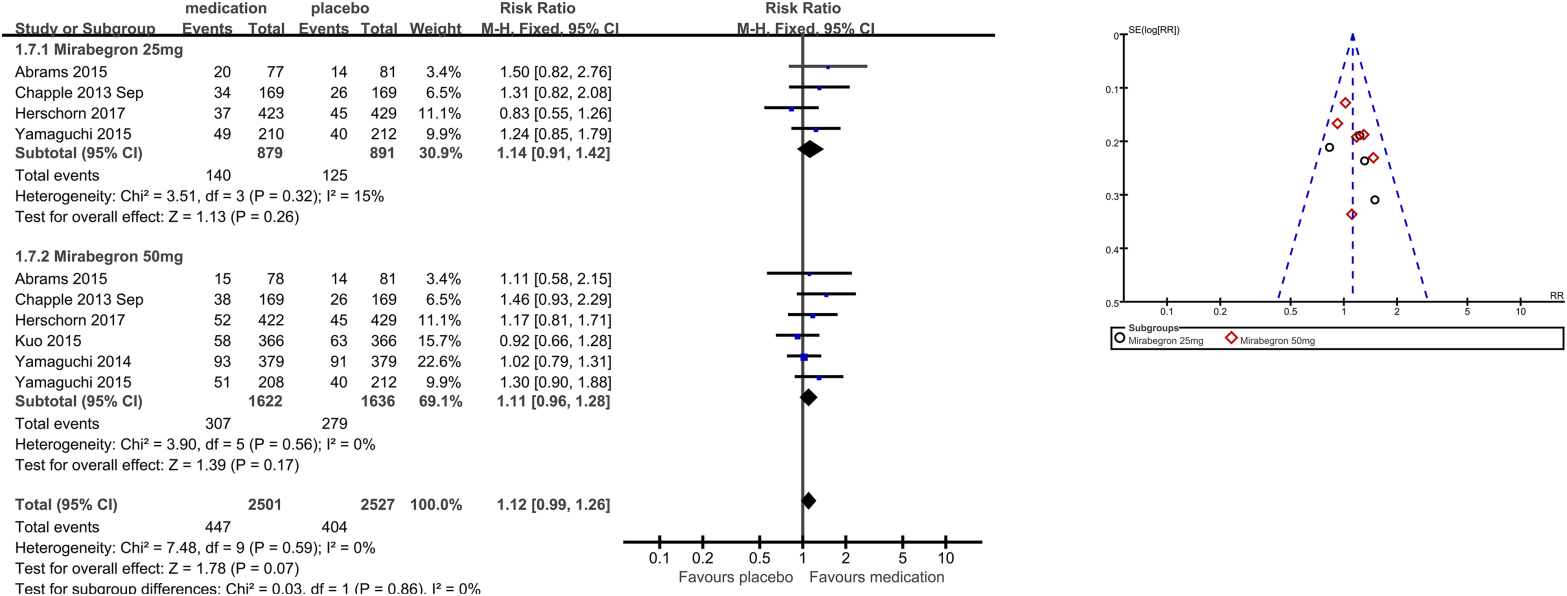
Comparison 1. Mirabegron versus placebo, Outcome 1.7, TRAEs.

The number of people for TRAEs (Figure 9) in six parallel-group trials was reported (20, 21, 23, 26, 28, 29). There was no statistically significant difference for TRAEs between the mirabegron and placebo groups (RR 1.12, 95% CI 0.99–1.26, *P* = 0.07, Outcome 1.7).

Four trials (20, 21, 23, 29) reported available data for mirabegron 25 mg in TRAEs (RR 1.14, 95% CI 0.91–1.42, *P* = 0.26, Outcome 1.7.1). Six trials (20, 21, 23, 26, 28, 29) reported available data for mirabegron 50 mg in TRAEs, with no statistically significant difference (RR 1.11, 95% CI 0.96–1.28, *P* = 0.17, Outcome 1.7.2).

**Figure 10:**
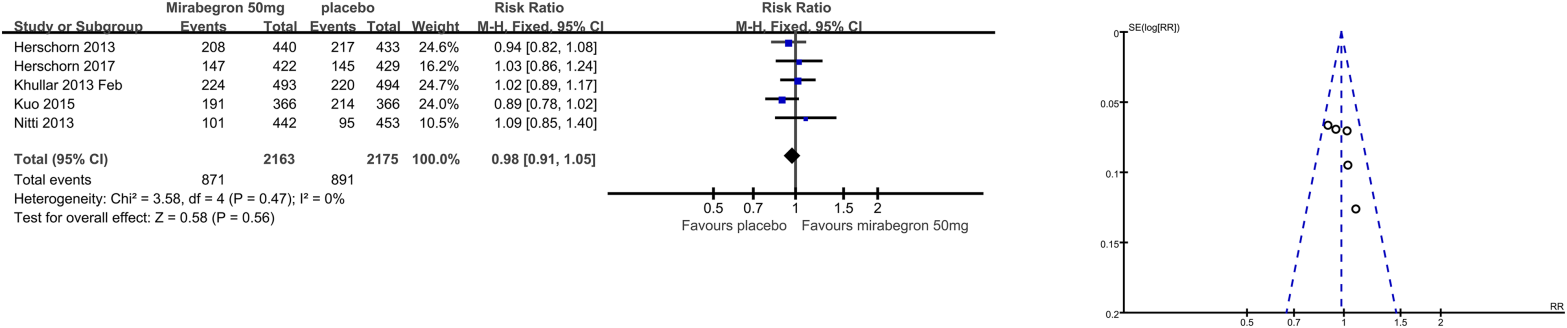
Comparison 1. Mirabegron versus placebo, Outcome 1.8, TEAEs.

The number of people for TEAEs (Figure 10) in five parallel-group trials was reported (22, 23, 25–27). There was no statistically significant difference for TEAEs between the mirabegron and placebo groups (RR 0.98, 95% CI 0.91–1.05, *P* = 0.56, Outcome 1.8).

Despite the clinical heterogeneity of the included studies (such as demographics), from the statistical tests, we considered heterogeneity to be acceptable for *I*^2^ <50% (referenced in the Cochrane Handbook of Systematic Evaluation of Interventions). GRADEprofiler Version 3.6 was used to evaluate the quality of the evidence for the summarized findings. The results of the quality of evidence grading are shown in the “Summary of findings” presented in the Appendix.

## Discussion

This article is one of a series of articles on β_3_-adrenergic receptor agonist mirabegron therapy for overactive bladder symptoms, and it should be viewed in that context. The use of mirabegron for the relief of overactive bladder symptoms is widespread, so the question of which dose of mirabegron is better is of clinical interest. The two questions addressed by the article are as follows: whether mirabegron is better than placebo, and what dose is most effective and secure?

### Summary of main results

Considering this evaluation as a whole, mirabegron was found to be more effective than placebo for adults with overactive bladder syndrome. The difference in quantification of symptoms between the mirabegron and placebo groups was approximately 13 ml more volume voided per micturition (MD 12.50, 95% CI 10.72–14.28, *P* < 0.00001), five fewer micturitions per week (MD for micturitions within a day −0.60, 95% CI −0.70 to −0.50, *P* < 0.00001), and four fewer incontinence episodes per week (MD for incontinence episodes within a day −0.47, 95% CI −0.56 to −0.38, *P* < 0.00001) in favor of mirabegron. The difference in patients’ satisfaction scores with treatments between the mirabegron and placebo groups was approximately 1 score higher for TS-VAS (MD 0.78, 95% CI 0.59–0.97, *P* < 0.00001), 0.2 scores lower for PPBC (MD −0.14, 95% CI −0.25 to −0.03, *P* = 0.02), and 5 scores lower for OAB-q (MD −4.31, 95% CI −5.49 to −3.13, *P* < 0.00001) in favor of mirabegron. One in five people (Events/Total = 447/2,501) taking mirabegron reported TRAEs; the risk of discontinuation due to TRAEs was similar in the mirabegron and placebo groups (RR 1.12, 95% CI 0.99–1.26, *P* = 0.07), and the risk of TEAEs was also similar to that in the placebo group (RR 0.98, 95% CI 0.91–1.05, *P* = 0.56). As noted earlier, there was no significant tendency for mirabegron to be associated with overall adverse events compared with placebo, so its safety profile was relatively favorable.

Doses higher and lower than the normal therapeutic dose of 50 mg once daily, which is 25 mg vs. 100 mg of mirabegron, were indirectly compared by examining the combined statistics and the test for subgroup differences for each dose of mirabegron vs. placebo. Test for subgroup differences in volume voided per micturition was the statistically significant difference [*χ*^2 ^= 8.13, df = 2 (*p* = 0.02), *I*^2^ 75.4%]. Mirabegron 50 mg (MD 13.41, 95% CI 11.08–15.75, *P* < 0.00001) demonstrated superior efficacy in volume voided per micturition when compared to mirabegron 25 mg (MD 7.51, 95% CI 3.58–11.44, *P* = 0.0002); however, there was similar efficacy when 100 mg (MD 14.78, 95% CI 10.94–18.62, *P* < 0.00001) of mirabegron was compared to mirabegron 50 mg. A 50 mg dose made no difference between 25 mg and 100 mg for decreasing micturitions [*χ*^2 ^= 1.14, df = 2 (*p* = 0.57), *I*^2^ 0%] and incontinence episodes [*χ*^2 ^= 0.23, df = 2 (*p* = 0.89), *I*^2^ 0%] per 24 h. Test for subgroup differences in OAB-q was the statistically significant difference [*χ*^2 ^= 2.95, df = 1 (*p* = 0.09), *I*^2^ 66.1%]. Patient-reported reductions in OAB-q were significantly better with larger doses, which were 50 mg (MD −4.95, 95% CI −6.33 to −3.56, *P* < 0.00001) superior to 25 mg (MD −2.64, 95% CI −4.88 to −0.40, *P* = 0.02). Because the risk of TRAEs was similar [*χ*^2^ = 0.03, df = 1 (*p* = 0.86), *I*^2^ 0%] for mirabegron 25 mg (Events/Total = 140/879) and mirabegron 50 mg (Events/Total = 307/1,622), patients tolerated mirabegron better. Only 25 mg and 50 mg are available commercially. Based on a comprehensive analysis of the data, including combined statistics, 95% CI, and weights, the recommended dose of 50 mg is preferable as it balances the significance, stability, and safety of efficacy and therefore has greater generalizability to support policymakers in promoting it.

During normal filling, an increase in the volume of the bladder does not cause a significant increase in its internal pressure. It is when the volume of the bladder is >300–400 ml that its internal pressure rises significantly, at which point the receptors on the bladder wall and in the posterior urethra are stimulated by stretching and become excited. This excitation travels along the afferent fibers of the pelvic nerve to the sacral segment of the spinal cord and then up the brainstem and cerebral cortex to produce the urge to urinate. Overactive bladder syndrome is a condition in which the bladder suddenly contracts without any control, resulting in urination and/or leakage of urine. It is also known as “irritable” bladder or detrusor instability, urgency to urinate, and/or urgency incontinence syndrome. Overactive bladder syndrome becomes more common with age. The functional regulation of the detrusor muscle of the bladder is accomplished by a variety of factors such as cholinergic nerves, adrenergic nerves, non-cholinergic and non-adrenergic nerves, and the detrusor muscle itself. The myogenic and neurogenic hypotheses are the two most frequently recognized explanations for OAB, while its pathophysiology is still not completely understood. The detrusor muscle grows overactive in both hypotheses (10). Mirabegron is a β_3_-adrenergic receptor agonist that selectively stimulates bladder β_3_-adrenergic receptors, mediates relaxation of the detrusor, and modulates sensory pathways, bladder afferent neural activity, and neurotransmitter release, from the urothelium, thereby increasing bladder capacity and decreasing bladder sensitivity to alleviate the storage-phase symptom syndrome (16, 17). At the same time, mirabegron has a concentration-dependent diastolic effect on the detrusor, with high concentrations of mirabegron acting synergistically to diastole the detrusor by agonizing the β_3_-adrenergic receptor and antagonizing the α_1_-adrenergic receptor (18). Herein lies the potential reason for the superiority of mirabegron 50 mg over mirabegron 25 mg. The primary endpoint was assessed after 12 weeks of therapy in the majority of the included trials. Given that mirabegron is not curative for overactive bladder syndrome, which is a chronic illness, and it is not clear whether any benefits are sustained after treatment stops, regular usage and long-term adherence to the medication are probably necessary to sustain the benefits.

### Quality of the evidence

Since 2012, when mirabegron was approved by the US Food and Drug Administration for the treatment of OAB symptoms, there have been a significant number of trials examining the efficacy and security of mirabegron in the treatment of OAB symptoms. Generally speaking, the reported methods of the parallel arm trials were of moderate to high quality. Nevertheless, the methods of group allocation were rarely described in enough detail to guarantee that the allocation was sufficiently concealed. Only two of the nine double-blinded trials explicitly indicated that outcome assessors were unaware of group allocation. Subgroup allocation and reasons for withdrawal from the trials were fully reported in all but one of the nine trials.

### Potential biases in the evaluation process

It is sad that we focused only on these outcome metrics of interest and could not combine data on the additional outcomes reported in the nine trials. There are two reasons for this, one being the limited energy of those involved in this evaluation and the other key factor being that both the outcomes that were chosen and the way that the same outcome was measured and reported varied.

All trials involved both men and women; however, there was no sex-specific reporting of results. Investigating gender-based disparities in effect was therefore not practicable. There was statistically significant heterogeneity in certain comparisons. A reasonable explanation based on clinical heterogeneity is typically available for this. The sample populations varied, but there were also variations in the ways that drugs were administered.

It is important to note that every trial explicitly stated pharmaceutical company support. This aid included everything from full funding, data analysis, and help with medical writing to the design and execution of the trial, the provision of active and placebo tablets (in blinded packaging), and more.

## Authors’ conclusions

### Implications for practice

Statistically significant differences are observed when mirabegron is administered for the treatment of overactive bladder syndrome in comparison to a placebo. Patients who received mirabegron therapy were more likely to report a cure or improvement in their symptoms, as well as an increase in the volume passed (approximately thirteen ml per micturition), a decrease in the frequency of micturitions (about five per week), and a decrease in the frequency of incontinence episodes (about four per week). In terms of satisfaction with treatments including TS-VAS, PPBC, and OAB-q, it has also improved appreciably. About one in five people taking mirabegron reported TRAEs. There was no significant drug predisposition for the risk of TRAEs and TEAEs compared to the placebo group, resulting in a favorable safety profile for mirabegron therapy. Mirabegron 50 mg was more advantageous in increasing volume voided per micturition, reducing OAB-q; however, the risk of TRAEs occurring was similar to the lower dose and was therefore well tolerated. The effect is maximized by taking 50 mg once daily for a long period of time.

### Implications for research

The majority of the trials that were included used oral pill delivery. Further study would be beneficial to see whether variations in the size of the effect with various delivery methods (such as skin patches, OCAS formulation, or intravesical administration) would also be beneficial (30). Because it delivers the medication directly to the site of action, intravesical administration has the potential to eliminate some of the difficult side effects of 3 adrenergic agonists. However, this method would only be therapeutically helpful if intravesical administration could be made less difficult. In addition, very few trials have involved high doses of mirabegron (100 mg, 150 mg, 200 mg) in their studies, and future trials are needed to assess the efficacy and safety of these doses.

Mirabegron is unlikely to be curative; continued use of it will probably be necessary for success. Little is known about the forward effect and acceptance of mirabegron therapy because of the lack of longer follow-up (5 years, 10 years, or more) in the majority of trials. Although it wasn't a requirement in every experiment, patient satisfaction and therapy acceptance are crucial considerations in management decisions. This information will need to be known through follow-up in the future.

## Data Availability

The original contributions presented in the study are included in the article/Supplementary Material, further inquiries can be directed to the corresponding authors.

## Appendix

**Characteristics of studies.**

Characteristics of included studies *(ordered by study ID)*

Abrams et al. (20)

**Table:**

Methods	RCT. Placebo and monotherapy controlled, parallel design Phase II Double-blind Multicenter (141) and multinational (20) Masking of assessors not stated
Participants	1,306 patients Inclusion criteria: male and female patients aged ≥18 years with symptoms of OAB for ≥3 months. Following a 2-week, single-blind placebo run-in period and washout of existing OAB medications (prior use of solifenacin or mirabegron was not excluded) and prohibited medications, patients with eight or more micturitions per 24 h and one urgency episode or more per 24 h (with or without incontinence), based on a 3-day electronic patient micturition diary Exclusion criteria: not clear
Interventions	Group 1: placebo (*n* = 81) Group 2: mirabegron 25 mg qd (*n* = 77) Group 3: mirabegron 50 mg qd (*n* = 78) Group 4: solifenacin 2.5 mg qd (*n* = 79) Group 5: solifenacin 5 mg qd (*n* = 156) Group 6: solifenacin 10 mg qd (*n* = 78) Group 7: solifenacin 2.5 mg + mirabegron 25 mg qd (*n* = 149) Group 8: solifenacin 2.5 mg + mirabegron 50 mg qd (*n* = 149) Group 9: solifenacin 5 mg + mirabegron 25 mg qd (*n* = 144) Group 10: solifenacin 5 mg + mirabegron 50 mg qd (*n* = 153) Group 11: solifenacin 10 mg + mirabegron 25 mg qd (*n* = 81) Group 12: solifenacin 10 mg + mirabegron 50 mg qd (*n* = 81) Twelve-week treatment period Two-week run-in
Outcomes	MVV, mean number of micturitions/24 h, mean number of incontinence episodes/24 h, mean number of urgency episodes/24 h Laboratory assessments Blood pressure and pulse rate ECG, PVR TEAEs
Notes	Abstract Method of randomization not described 67 dropouts (group not stated) Reasons for discontinuation mentioned Two-week follow-up Company support declared

**Table:** Risk of bias

Bias	Authors’ judgment	Support for judgment
Random sequence generation	Low risk	Adequate
Allocation concealment	Low risk	Adequate
Blinding of participants and personnel	Low risk	Adequate
Blinding of outcome assessment	Unclear risk	Unclear
Incomplete outcome data	Low risk	Adequate
Selective reporting	Low risk	Adequate
Other bias	Unclear risk	Unclear

Chapple et al. (21)

**Table:**

Methods	RCT. Placebo and active controlled, parallel design Phase II Double-blind Multicenter and multinational Masking of assessors not stated
Participants	928 patients Inclusion criteria: men and women aged ≥18 years experiencing symptoms of OAB for ≥3 months with frequency of micturition on average ≥8 times per 24 h and at least three episodes of urgency (Grade 3 or 4), with or without incontinence, during a 3-day micturition diary period at baseline Exclusion criteria: clinically significant bladder outflow obstruction; significant PVR volume (>200 ml); incontinence where stress was the predominant factor; indwelling catheters or intermittent self-catheterization; diabetic neuropathy; symptomatic urinary tract infection, interstitial cystitis, bladder stones, previous pelvic radiation therapy or previous or current malignant disease of the pelvic organs; contraindications for anticholinergics; non-drug treatment, including electrostimulation therapy (although bladder training or pelvic floor exercise programs that had started more than 1 month prior to the start of the study could be continued); use of other urinary incontinence medications; known or suspected hypersensitivity to tolterodine, other anticholinergics, mirabegron, lactose, or any of the excipients; clinically significant cardiovascular (including ECG abnormalities) or cerebrovascular disease; or any other condition making the patient unsuitable for the study (as deemed by the investigator)
Interventions	Group 1: placebo (*n* = 166) Group 2: mirabegron 25 mg qd (*n* = 167) Group 3: mirabegron 50 mg qd (*n* = 167) Group 4: mirabegron 100 mg qd (*n* = 168) Group 5: mirabegron 200 mg qd (*n* = 166) Group 6: tolterodine 4 mg qd (*n* = 85) Twelve-week treatment period Two-week run-in
Outcomes	Mean number of micturitions/24 h, mean volume voided per micturition, mean number of urinary incontinence, urgency urinary incontinence, and urgency episodes/24 h Severity of urgency; number of nocturia episodes Changes in ICIQ-OAB and ICIQ-OABqol symptom scores Patients’ perception of treatment benefit Incidence and severity of adverse events Vital signs, laboratory tests, ECG, PVR
Notes	Abstract Method of randomization not described 196 dropouts (Group 1, 12; Group 2, 16; Group 3, 16; Group 4, 7; Group 5, 16; Group 6, 3) Reasons for discontinuation mentioned No follow-up Company support declared

**Table:** Risk of bias

Bias	Authors’ judgment	Support for judgment
Random sequence generation	Low risk	Adequate
Allocation concealment	Low risk	Adequate
Blinding of participants and personnel	Low risk	Adequate
Blinding of outcome assessment	Unclear risk	Unclear
Incomplete outcome data	Low risk	Adequate
Selective reporting	Low risk	Adequate
Other bias	Unclear risk	Unclear

Herschorn et al. (22)

**Table:**

Methods	RCT. Placebo controlled, parallel design Phase Ⅲ Double-blind Multicenter (151) and multinational Masking of assessors not stated
Participants	1,306 patients Inclusion criteria: patients aged ≥18 years with OAB symptoms for ≥3 months were enrolled in a 2-week, single-blind, placebo run-in. Over a 3-day micturition diary period, patients with an average of ≥8 micturitions per 24 h and ≥3 urgency episodes (Grade 3 or 4 on the five-point patient perception of intensity of urgency scale (0 ¼, no urgency; 1 ¼, mild urgency; 2 ¼, moderate urgency; 3 ¼, severe urgency; 4 ¼, urge incontinence), with or without incontinence Exclusion criteria: average total daily urine volume of >3,000 ml during the diary period and significant stress incontinence or mixed stress or urge incontinence, where stress was the predominant factor
Interventions	Group 1: placebo (*n* = 433) Group 2: mirabegron 25 mg qd (*n* = 432) Group 3: mirabegron 50 mg qd (*n* = 440) Twelve-week treatment period Two-week run-in
Outcomes	Mean number of incontinence episodes and micturitions/24 h Mean volume voided per micturition, mean number of incontinence episodes and micturitions/24 h Mean level of urgency, number of urgency incontinence episodes and urgency (Grade 3 or 4) episodes/24 h OAB-q, TS-VAS, PPBC
Notes	Abstract Method of randomization not described 196 dropouts (Group 1, 46; Group 2, 54; Group 3, 66) Reasons for discontinuation mentioned No follow-up Company support declared

**Table:** Risk of bias

Bias	Authors’ judgment	Support for judgment
Random sequence generation	Low risk	Adequate
Allocation concealment	Low risk	Adequate
Blinding of participants and personnel	Low risk	Adequate
Blinding of outcome assessment	Unclear risk	Unclear
Incomplete outcome data	Low risk	Adequate
Selective reporting	Low risk	Adequate
Other bias	Unclear risk	Unclear

Herschorn et al. (23) and Robinson et al. (24)

**Table:**

Methods	RCT. Placebo and active controlled, parallel design Phase Ⅲ Double-blind Multicenter (435) and multinational(42) Masking of assessors not stated
Participants	3,527 patients Inclusion criteria: patients aged ≥18 years with symptoms of OAB for ≥3 months who recorded on average ≥8 micturition episodes/24 h, ≥1 urgency episode/24 h, and ≥3 incontinence episodes over a 7-day period prior to randomization Exclusion criteria: the presence of an indwelling catheter; chronic inflammation due to bladder pain syndrome or interstitial cystitis; intravesical treatment in the previous 12 months; urinary or gastric retention; severe ulcerative colitis; any contraindication against antimuscarinic agents; significant cardiovascular or cerebrovascular diseases within 6 months of screening; QT prolongation; severe hypertension (defined as SBP ≥ 180 mmHg and/or average DBP ≥ 110 mmHg when sitting); moderate-to-severe hepatic impairment; severe renal impairment; known hypersensitivity to solifenacin or mirabegron; post-void residual volume of >150 ml; significant mixed-urinary incontinence where stress urinary incontinence was the predominant feature
Interventions	Group 1: placebo (*n* = 429) Group 2: mirabegron 25 mg qd (*n* = 423) Group 3: mirabegron 50 mg qd (*n* = 422) Group 4: solifenacin 5 mg qd (*n* = 423) Group 5: solifenacin 5 mg + mirabegron 25 mg qd (*n* = 853) Group 6: solifenacin 5 mg + mirabegron 50 mg qd (*n* = 848) Twelve-week treatment period Four-week run-in Two-week run-out
Outcomes	Mean number of UI episodes/24 h and micturitions/24 h Mean volume voided/micturition PROs: OAB-q, TS-VAS, PPBC, HRQoL TEAEs PVR, laboratory parameters
Notes	Abstract Method of randomization not described 341 dropouts (Group 1, 43; Group 2, 44; Group 3, 50; Group 4, 37; Group 5, 82; Group 6, 85) Reasons for discontinuation mentioned No follow-up Company support declared

**Table:** Risk of bias

Bias	Authors’ judgment	Support for judgment
Random sequence generation	Low risk	Adequate
Allocation concealment	Low risk	Adequate
Blinding of participants and personnel	Low risk	Adequate
Blinding of outcome assessment	Unclear risk	Unclear
Incomplete outcome data	Low risk	Adequate
Selective reporting	Low risk	Adequate
Other bias	Unclear risk	Unclear

Khullar et al. (25)

**Table:**

Methods	RCT. Placebo and active controlled, parallel design Phase Ⅲ Double-blind Multicenter (189) and multinational (27) Masking of assessors not stated
Participants	1,987 patients Inclusion criteria: men and women aged ≥18 years with symptoms of OAB for ≥3 months. An average micturition frequency of eight or more times per 24 h period and at least three episodes of urgency, with or without incontinence, during a 3-day micturition diary period Exclusion criteria: stress incontinence or stress-predominant mixed incontinence at screening or an average total daily urine volume of >3,000 ml as recorded in a 3-day micturition diary period
Interventions	Group 1: placebo (*n* = 494) Group 2: mirabegron 50 mg qd (*n* = 493) Group 3: mirabegron 100 mg qd (*n* = 496) Group 4: tolterodine 4 mg qd (*n* = 495) Twelve-week treatment period Two-week run-in
Outcomes	Mean number of incontinence episodes and micturitions/24 h. Mean volume voided per micturition, mean number of incontinence episodes, and micturitions/24 h. QoL: OAB-q, PPBC, TS-VAS Reporting of adverse events Clinical laboratory assessments Vital signs, physical examination ECG, PVR
Notes	Abstract 196 dropouts (Group 1, 44; Group 2, 57; Group 3, 45; Group 4, 50) Reasons for discontinuation mentioned Telephone or visit for 30 days of follow-up Company support declared

**Table:** Risk of bias

Bias	Authors’ judgment	Support for judgment
Random sequence generation	Low risk	Adequate
Allocation concealment	Low risk	Adequate
Blinding of participants and personnel	Low risk	Adequate
Blinding of outcome assessment	Low risk	Adequate
Incomplete outcome data	Low risk	Adequate
Selective reporting	Low risk	Adequate
Other bias	Unclear risk	Unclear

Kuo et al. (26)

**Table:**

Methods	RCT. Placebo and active controlled, parallel design Phase Ⅲ Double-blind Multicenter (67) and multinational Masking of assessors not stated
Participants	1,126 patients Inclusion criteria: male and female outpatients who met the legal minimum age requirement of the region [18 years old, China and India; 20 years old, Korea (at the time of the study) and Taiwan] and who had symptoms of OAB for ≥3 months. Symptoms of OAB for at least 12 weeks before initiation of the run-in period; an average of ≥8 micturitions/24 h; an average of ≥1 episode of urgency or urgency incontinence/24 h, during a 3-day micturition diary period Exclusion criteria: stress urinary incontinence as a predominant symptom at screening; urinary tract infection, urinary stone, interstitial cystitis, or a history of recurrent urinary tract infection; confirmed PVR volume of ≥100 ml or a clinically significant lower urinary tract obstructive disease; an average total daily urine volume of >3,000 ml (as recorded in a 3-day voiding diary period); uncontrolled hypertension (sitting systolic blood pressure of ≥180 mmHg or diastolic blood pressure of ≥110 mmHg); pulse rate of ≥110 beats per minute (bpm) or <50 bpm; subject has indwelling catheter or practices intermittent self-catheterization
Interventions	Group 1: placebo (*n* = 323) Group 2: mirabegron 50 mg qd (*n* = 338) Group 3: tolterodine 4 mg qd (*n* = 333) Twelve-week treatment period Two-week run-in
Outcomes	Mean number of micturitions/24 h Mean number of urgency episodes, urinary incontinence episodes, urgency incontinence episodes, and nocturia episodes/24 h Mean volume voided/micturition QoL: the King's Health Questionnaire (KHQ) Adverse events Clinical laboratory assessments Vital signs, physical examination ECG, PVR
Notes	Abstract 205 dropouts (Group 1, 77; Group 2, 61; Group 3, 67) Reasons for discontinuation mentioned Two-week follow-up Company support declared

**Table:** Risk of bias

Bias	Authors’ judgment	Support for judgment
Random sequence generation	Low risk	Adequate
Allocation concealment	Low risk	Adequate
Blinding of participants and personnel	Low risk	Adequate
Blinding of outcome assessment	Unclear risk	Unclear
Incomplete outcome data	Low risk	Adequate
Selective reporting	Low risk	Adequate
Other bias	Unclear risk	Unclear

Nitti et al. (27)

**Table:**

Methods	RCT. Placebo controlled, parallel design Phase Ⅲ Double-blind Multicenter (132) Masking of assessors not stated
Participants	1,329 patients Inclusion criteria: male and female patients aged 18 years or older were screened for enrollment in the study if they had OAB symptoms for 3 or more months. At baseline, patients must have experienced an average of 8 or more micturitions per 24 h and 3 or more urgency episodes (Grade 3, severe urgency; Grade 4, urge incontinence) with or without incontinence during a 3-day period and must have continued to meet all screening eligibility criteria. Exclusion criteria: patients were excluded from study if they had clinically relevant stress incontinence or mixed stress/urgency incontinence with stress as the predominant factor; an indwelling catheter; evidence of a symptomatic urinary tract infection, chronic inflammation, bladder stones, previous pelvic radiation therapy, or previous or current malignant disease of the pelvic organs; severe hypertension (sitting average SBP of 180 mmHg or greater and/or average DBP of 110 mmHg or greater); or use of OAB medications which could not be stopped safely at screening
Interventions	Group 1: placebo (*n* = 453) Group 2: mirabegron 50 mg qd (*n* = 442) Group 3: mirabegron 100 mg qd (*n* = 433) Twelve-week treatment period Two-week run-in.
Outcomes	Mean number of incontinence episodes/24 h and mean number of micturitions/24 h Mean volume voided per micturition and mean numbers of incontinence episodes/24 h and micturitions/24 h Mean levels of urgency, number of urgency incontinence episodes/24 h, and number of Grade 3/4 urgency episodes/24 h OAB-q, TS-VAS, PPBC
Notes	Abstract One patient did not participate in the double-blind study drug 181 dropouts (Group 1, 69; Group 2, 59; Group 3, 53) Reasons for discontinuation mentioned Telephone or visit for 30 days of follow-up Company support declared

**Table:** Risk of bias

Bias	Authors’ judgment	Support for judgment
Random sequence generation	Low risk	Adequate
Allocation concealment	Low risk	Adequate
Blinding of participants and personnel	Low risk	Adequate
Blinding of outcome assessment	Unclear risk	Unclear
Incomplete outcome data	Low risk	Adequate
Selective reporting	Low risk	Adequate
Other bias	Unclear risk	Unclear

Yamaguchi et al. (28)

**Table:**

Methods	RCT. Placebo controlled, parallel design Phase Ⅲ Double-blind Multicenter
Participants	1,139 patients Inclusion criteria: men or women aged ≥20 years, with OAB symptoms for ≥24 weeks. Patients with an average of ≥8 micturitions/24 h and ≥1 urgency episode/24 h and/or ≥1 urgency incontinence episode/24 h, confirmed using 3-day micturition diaries Exclusion criteria: key OAB-related exclusion criteria included a diagnosis of genuine stress incontinence, an average total daily urine volume of >3,000 ml during the 3-day pretreatment micturition diary period, and a post-void residual urine volume of at least 100 ml when measured before treatment
Interventions	Group 1: placebo (*n* = 368) Group 2: mirabegron 50 mg qd (*n* = 369) Group 3: tolterodine 4 mg qd (*n* = 368) Twelve-week treatment period Two-week run-in
Outcomes	Mean number of micturitions/24 h; number of urgency episodes/24 h; number of incontinence episodes/24 h; number of urgency incontinence episodes/24 h; volume voided/micturition; number of nocturia episodes QoL: KHQ Adverse events Laboratory findings BP and pulse rate, ECG
Notes	Abstract Method of randomization not described More than 82% of patients were female. 85 dropouts (Group 1, 31; Group 2, 31; Group 3, 23) Reasons for discontinuation mentioned Two-week follow-up Company support declared

**Table:** Risk of bias

Bias	Authors’ judgment	Support for judgment
Random sequence generation	Low risk	Adequate
Allocation concealment	Low risk	Adequate
Blinding of participants and personnel	Low risk	Adequate
Blinding of outcome assessment	Unclear risk	Unclear
Incomplete outcome data	Low risk	Adequate
Selective reporting	Low risk	Adequate
Other bias	Unclear risk	Unclear

Yamaguchi et al. (29)

**Table:**

Methods	RCT. Placebo controlled, parallel design Phase Ⅱ Double-blind Multicenter
Participants	842 patients Inclusion criteria: male or female outpatients aged ≥20 years, with OAB symptoms for ≥24 weeks. Patients with an average of ≥8 micturitions/24 h and ≥1 urgency episode and/or ≥1 urgency incontinence episode/24 h, according to a 3-day micturition diary Exclusion criteria: patients with polyuria exceeding 3,000 ml in mean daily micturition volume and a clear diagnosis of stress incontinence
Interventions	Group 1: placebo (*n* = 211) Group 2: mirabegron 25 mg qd (*n* = 209) Group 3: mirabegron 50 mg qd (*n* = 208) Group 4: mirabegron 100 mg qd (*n* = 207) Twelve-week treatment period Two-week run-in
Outcomes	Mean number of micturitions/24 h; number of urgency episodes/24 h; number of incontinence episodes/24 h; number of urgency incontinence episodes/24 h; volume voided/micturition; number of nocturia episodes QoL: KHQ Adverse events Laboratory findings BP and pulse rate, ECG
Notes	Abstract Method of randomization not described More than 80% of patients were female. 53 dropouts (Group 1, 16; Group 2, 11; Group 3, 13; Group 4, 13) Reasons for discontinuation mentioned No follow-up Company support declared

**Table:** Risk of bias

Bias	Authors’ judgment	Support for judgment
Random sequence generation	Low risk	Adequate
Allocation concealment	Low risk	Adequate
Blinding of participants and personnel	Low risk	Adequate
Blinding of outcome assessment	Low risk	Adequate
Incomplete outcome data	Low risk	Adequate
Selective reporting	Low risk	Adequate
Other bias	Unclear risk	Unclear
mo, months; h/hr, hours; BP, blood pressure; HRQoL, health-related quality of life; QT, electrocardiogram QT; SBP, systolic blood pressure; DBP, diastolic blood pressure; bpm, beats per minutes; mmHg, millimeter of mercury; ml, milliliter; OAB-q, overactive bladder questionnaire; TS-VAS, treatment satisfaction visual scale; PPBC, patient perception of bladder condition; ICIQ, International Consultation on Incontinence Questionnaire; TEAEs, treatment-emergent adverse events; PVR, postvoid residual; MVV, mean volume voided per micturition; ECG, electrocardiogram; mg, milligram; OAB, overactive bladder; qd, one time per day; QoL, quality of life; RCT, randomized control trial; PROs, patient-reported outcomes; UI, urinary incontinence; KHQ the King's Health Questionnaire.		

**Table:**

Study	Reason for exclusion
Abrams et al. (31)	No usable data in the abstract
Chapple et al. (32)	The study was not placebo controlled
Chapple et al. (33)	Treatment was given for 4 weeks and <12 weeks
Chapple et al. (34)	A pooled analysis of four studies. Trials not reported separately
Castro-Diaz et al. (35)	A pooled analysis of three studies. Trials not reported separately
Chen and Kuo (36)	No usable data in the abstract
Chapple et al. (37)	A large comprehensive clinical trial database analysis. Trials not reported separately
Cho et al. (38)	The duration of placebo-controlled treatment was 8 weeks and <12 weeks
Chen et al. (39)	This study investigated the efficacy and adverse events of mirabegron and solifenacin for managing overactive bladder syndrome in Sjogren syndrome. The study was not placebo controlled
Drake et al. (40)	The study was not placebo controlled
Drake et al. (41)	The study was not placebo controlled
Eltink et al. (42)	An open-label, cross-sectional study. Healthy male and female volunteers in the study
Griebling (43)	An editorial comment. The duration of treatment in the study was 6 weeks and <12 weeks
Gibson et al. (44)	There was no placebo lead period. The study was not placebo controlled
Gratzke et al. (45)	The study was not placebo controlled
Griebling et al. (46)	RCT. The aim was to evaluate the effect of mirabegron on the cognitive function of elderly patients with overactive bladder
Hsiao et al. (47)	Participants were only female OAB patients. Micturition episodes/72 h and urgency episodes/72 h were assessed
Herschorn et al. (48)	This was an 8-week crossover study
Hsiao et al. (49)	The aim was to elucidate the impact of mirabegron versus solifenacin on autonomic function and peripheral arterial conditions in women with OAB. The study was not placebo controlled
Huang et al. (50)	The trial was designed to examine the change in composite cognitive function 24 weeks after initiation of treatments in older ambulatory women with urgency-predominant incontinence
Inoue and Yokoyama (51)	A prospective randomized crossover study
Illiano et al. (52)	No usable data in the abstract. The study was not placebo controlled
Ito et al. (53)	The study was not placebo controlled
Khullar et al. (54)	A *post hoc* analysis of a randomized European–Australian Phase 3 trial
Kosilov et al. (55)	Treatment was given for 6 weeks and <12 weeks.
Krhut et al. (56)	This study included 78 patients suffering from spinal cord injury or multiple sclerosis. There was no adjusted mean change from the baseline
Krhut et al. (57)	RCT. Treatment was given for 4 weeks and <12 weeks. The aim was to evaluate the cardiovascular safety of mirabegron in the treatment of patients with neurogenic detrusor overactivity due to spinal cord injury or multiple sclerosis
Kinjo et al. (58)	The aim was to compare the efficacy and safety of mirabegron versus vibegron in postmenopausal women with treatment-naive OAB. The study was not placebo controlled
Liao and Kuo (59)	The study was not placebo controlled
Malik et al. (60)	The potential effects of the selective β_3_-adrenoceptor agonist mirabegron on cardiac repolarization were studied in healthy subjects. ECG was the only outcome
Mueller et al. (61)	The study was not placebo controlled
Moussa et al. (62)	Not found
Nakai et al. (63)	An open-labeled, randomized, non-placebo-controlled study
Otsuka et al. (64)	Comparison of mirabegron and imidafenacin for efficacy and safety. The study was not placebo controlled
Özkidik et al. (65)	The aim was to evaluate the efficacy and tolerability of mirabegron in the treatment of postsurgical bladder overactivity in women with stress urinary incontinence. A non-placebo-controlled study
Serati et al. (66)	This was an observational analytical prospective cohort study. The participants were women only. The study was not placebo controlled
Staskin et al. (67)	This was an 8-week crossover study.The study was not placebo controlled
Suzuki et al. (68)	A comparison of oxybutynin patches and mirabegron in the treatment of female patients with overactive bladder at 8 weeks. The study was not placebo controlled
Torimoto et al. (69)	A prospective randomized cross-over study. The study was not placebo controlled
Vecchioli Scaldazza and Morosetti (70)	No usable data in the abstract. The study was not placebo controlled
Wein (71)	An editorial comment.
Weber et al. (72)	The aim of this study was only to perform a BP safety evaluation in patients with an OAB
Welk et al. (73)	The patients with spinal cord injury (SCI) or multiple sclerosis (MS) with urinary symptoms and incontinence were recruited. A dose-escalation study on the same patient
Wagg et al. (74)	The study was designed to evaluate mirabegron in a flexible dosing regimen compared with placebo in a 12-week treatment period
Wang et al. (75)	The aim was to investigate whether adding an anticholinergic or β_3_-agonist can improve the therapeutic effect of intravesical onabotuliumtoxinA injection in patients with refractory OAB

Characteristics of excluded studies *(ordered by study ID)*

## Summary of findings

**Table:**

Mirabegron versus placebo for overactive bladder syndrome in adults						
Patient or population: patients with overactive bladder syndrome in adults Settings: Intervention: mirabegron vs. placebo						
Outcomes	Illustrative comparative risks* (95% CI)		Relative effect (95% CI)	No of participants (studies)	Quality of the evidence (GRADE)	Comments
	Assumed risk	Corresponding risk				
** **	Control	Mirabegron vs. placebo	** **	** **	** **	** **
**Mean volume voided per micturition** Follow-up: 0–4 weeks		The mean volume voided per micturition in the intervention groups was **12.5 higher** (10.72–14.28 higher)		10,882 (9 studies)	⊕⊕⊕⊕ **High**	
**Mean volume voided per micturition—mirabegron 25 mg** Follow-up: 0–2 weeks		The mean mean volume voided per micturition—mirabegron 25 mg in the intervention groups was **7.51 higher** (3.58–11.44 higher)		2,548 (5 studies)	⊕⊕⊕⊕ **High**	
**Mean volume voided per micturition—mirabegron 50 mg** Follow-up: 0–4 weeks		The mean mean volume voided per micturition—mirabegron 50 mg in the intervention groups was **13.41 higher** (11.08–15.75 higher)		5,780 (9 studies)	⊕⊕⊕⊕ **High**	
**Mean volume voided per micturition—mirabegron 100 mg** Follow-up: 0–4 weeks		The mean mean volume voided per micturition—mirabegron 100 mg in the intervention groups was **14.78 higher** (10.94–18.62 higher)		2,554 (4 studies)	⊕⊕⊕⊕ **High**	
**Micturitions in 24 h** Follow-up: 0–4 weeks		The mean micturition in 24 h in the intervention groups was **0.6 lower** (0.7–0.5 lower)		10,580 (8 studies)	⊕⊕⊕⊕ **High**	
**Micturitions in 24 h—mirabegron 25 mg**		The mean micturition in 24 h—mirabegron 25 mg in the intervention groups was **0.51 lower** (0.72–0.29 lower)		2,394 (4 studies)	⊕⊕⊕⊕ **High**	
**Micturitions in 24 h—mirabegron 50 mg** Follow-up: 0–4 weeks		The mean micturition in 24 h—mirabegron 50 mg in the intervention groups was **0.61 lower** (0.75–0.48 lower)		5,631 (8 studies)	⊕⊕⊕⊕ **High**	
**Micturitions in 24 h—mirabegron 100 mg** Follow-up: 0–4 weeks		The mean micturition in 24 h—mirabegron 100 mg in the intervention groups was **0.67 lower** (0.88–0.45 lower)		2,555 (4 studies)	⊕⊕⊕⊕ **High**	
**Incontinence episodes in 24 h** Follow-up: 0–4 weeks		The mean incontinence episodes in 24 h in the intervention groups was **0.47 lower** (0.56–0.38 lower)		8,287 (8 studies)	⊕⊕⊕⊕ **High**	
**Incontinence episodes in 24 h—mirabegron 25 mg**		The mean of incontinence episodes in 24 h—mirabegron 25 mg in the intervention groups was **0.48 lower** (0.67–0.3 lower)		1,954 (4 studies)	⊕⊕⊕⊕ **High**	
**Incontinence episodes in 24 h—mirabegron 50 mg** Follow-up: 0–4 weeks		The mean of incontinence episodes in 24 h—mirabegron 50 mg in the intervention groups was **0.45 lower** (0.57–0.33 lower)		4,240 (8 studies)	⊕⊕⊕⊕ **High**	
**Incontinence episodes in 24 h—mirabegron 100 mg** Follow-up: 0–4 weeks		The mean of incontinence episodes in 24 h—mirabegron 100 mg in the intervention groups was **0.5 lower** (0.69–0.31 lower)		2,093 (4 studies)	⊕⊕⊕⊕ **High**	
**TS-VAS** Follow-up: 0–4 weeks		The mean TS-VAS in the intervention groups was **0.78 higher** (0.59–0.97 higher)		3,350 (4 studies)	⊕⊕⊕⊕ **High**	
**PPBC** Follow-up: 0–4 weeks		The mean PPBC in the intervention groups was **0.14 lower** (0.25–0.03 lower)		2,559 (3 studies)	⊕⊕⊕⊕ **High**	
**OAB-q** Follow-up: 0–4 weeks		The mean OAB-q in the intervention groups was **4.31 lower** (5.49–3.13 lower)		5,729 (5 studies)	⊕⊕⊕⊕ **High**	
**OAB-q—mirabegron 25 mg**		The mean OAB-q—mirabegron 25 mg in the intervention groups was **2.64 lower** (4.88–0.4 lower)		1,937 (3 studies)	⊕⊕⊕⊕ **High**	
**OAB-q—mirabegron 50 mg** Follow-up: 0–4 weeks		The mean OAB-q—mirabegron 50 mg in the intervention groups was **4.95 lower** (6.33–3.56 lower)		3,792 (5 studies)	⊕⊕⊕⊕ **high**	
**TRAEs** Follow-up: 0–2 weeks	**Study population**		**RR 1.12** (0.99–1.26)	5,028 (6 studies)	⊕⊕⊕⊕ **High**	
	**160 per 1,000**	**179 per 1,000** (158–201)				
	**Moderate**					
	**173 per 1,000**	**194 per 1,000** (171–218)				
**TRAEs—mirabegron 25 mg** Follow-up: 0–2 weeks	**Study population**		**RR 1.14** (0.91–1.42)	1,770 (4 studies)	⊕⊕⊕⊕ **High**	
	**140 per 1,000**	**160 per 1,000** (128–199)				
	**Moderate**					
	**163 per 1,000**	**186 per 1,000** (148–231)				
**TRAEs—mirabegron 50 mg** Follow-up: 0–4 weeks	**Study population**		**RR 1.11** (0.96–1.28)	3,258 (6 studies)	⊕⊕⊕⊕ **High**	
	**171 per 1,000**	**189 per 1,000** (164–218)				
	**Moderate**					
	**173 per 1,000**	**192 per 1,000** (166–221)				
**TEAEs** Follow-up: 0–4 weeks	**Study population**		**RR 0.98** (0.91–1.05)	4,338 (5 studies)	⊕⊕⊕⊕ **High**	
	**410 per 1,000**	**401 per 1,000** (373–430)				
	**Moderate**					
	**445 per 1,000**	**436 per 1,000** (405–467)				
*The basis for the **assumed risk** (e.g., the median control group risk across studies) is provided in footnotes. The **corresponding risk** (and its 95% confidence interval) is based on the assumed risk in the comparison group and the **relative effect** of the intervention (and its 95% CI). **CI**, confidence interval; **RR**, risk ratio.						
GRADE Working Group grades of evidence. **High quality:** Further research is very unlikely to change our confidence in the estimate of effect. **Moderate quality:** Further research is likely to have an important impact on our confidence in the estimate of effect and may change the estimate. **Low quality:** Further research is very likely to have an important impact on our confidence in the estimate of effect and is likely to change the estimate. **Very low quality:** We are very uncertain about the estimate.						
